# P-676. Epidemiology and Burden of Respiratory Syncytial Virus in Hong Kong, 2013–2024: Insights into Temporal Trends, Risk Factors, and Clinical Outcomes

**DOI:** 10.1093/ofid/ofaf695.889

**Published:** 2026-01-11

**Authors:** Ophelia Wong, Ming Hong Choi, Fan Ngai Ivan Hung

**Affiliations:** The University of Hong Kong, Hong Kong, Hong Kong; Queen Mary Hospital, Hong Kong, Hong Kong; The University of Hong Kong, Hong Kong, Hong Kong

## Abstract

**Background:**

Respiratory syncytial virus (RSV) imposes a significant burden on global health, particularly among young children and older adults. Despite extensive global surveillance, granular epidemiological data from Hong Kong—a densely populated subtropical region—remain sparse. This study bridges this gap by analysing 11 years of population-level data, including the unprecedented impact of COVID-19 and post-pandemic resurgence.

**Figure:**
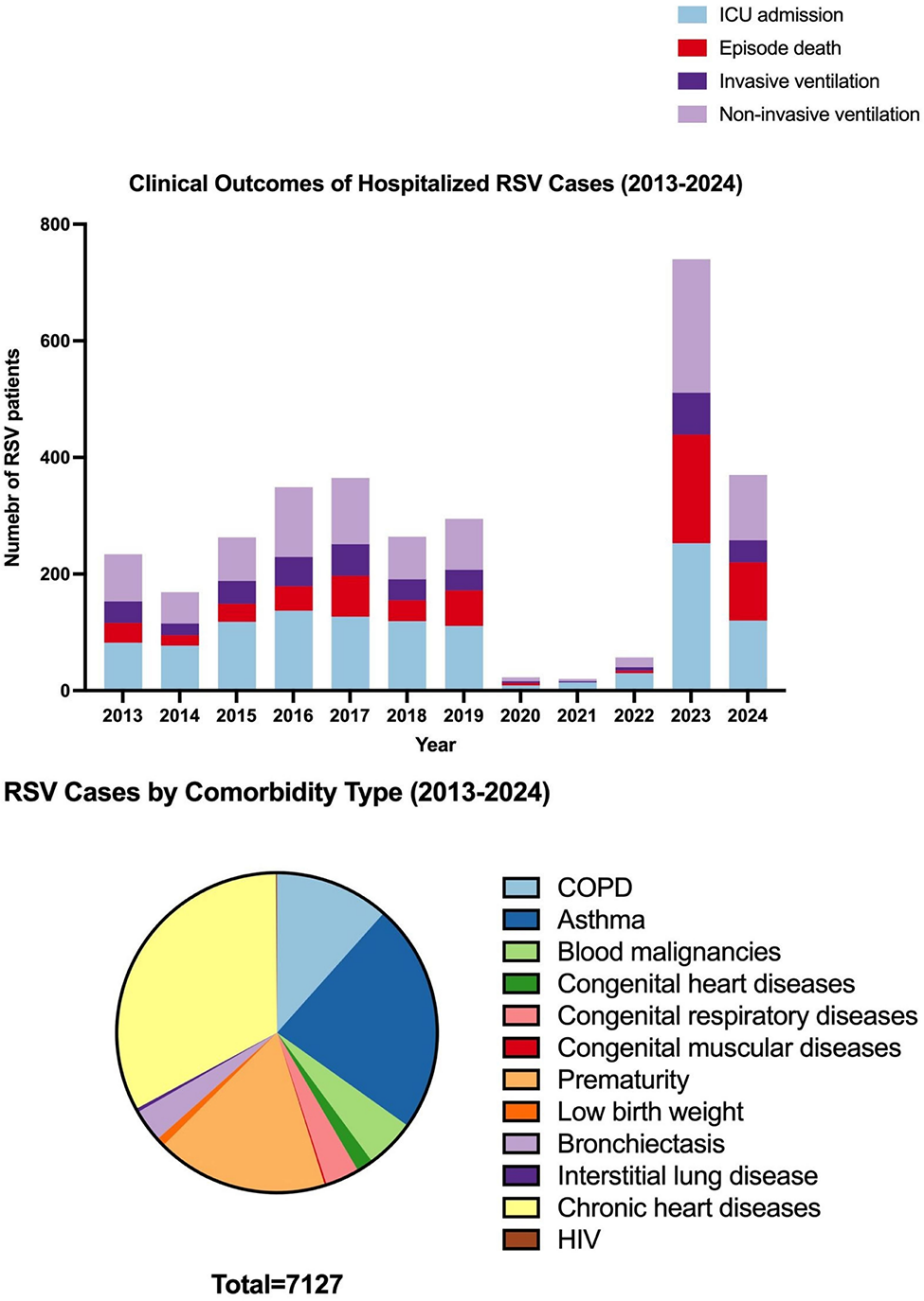
Clinical Outcomes of Hospitalized RSV Cases and RSV Cases by Comorbidity Type (2013-2024)

**Figure:**
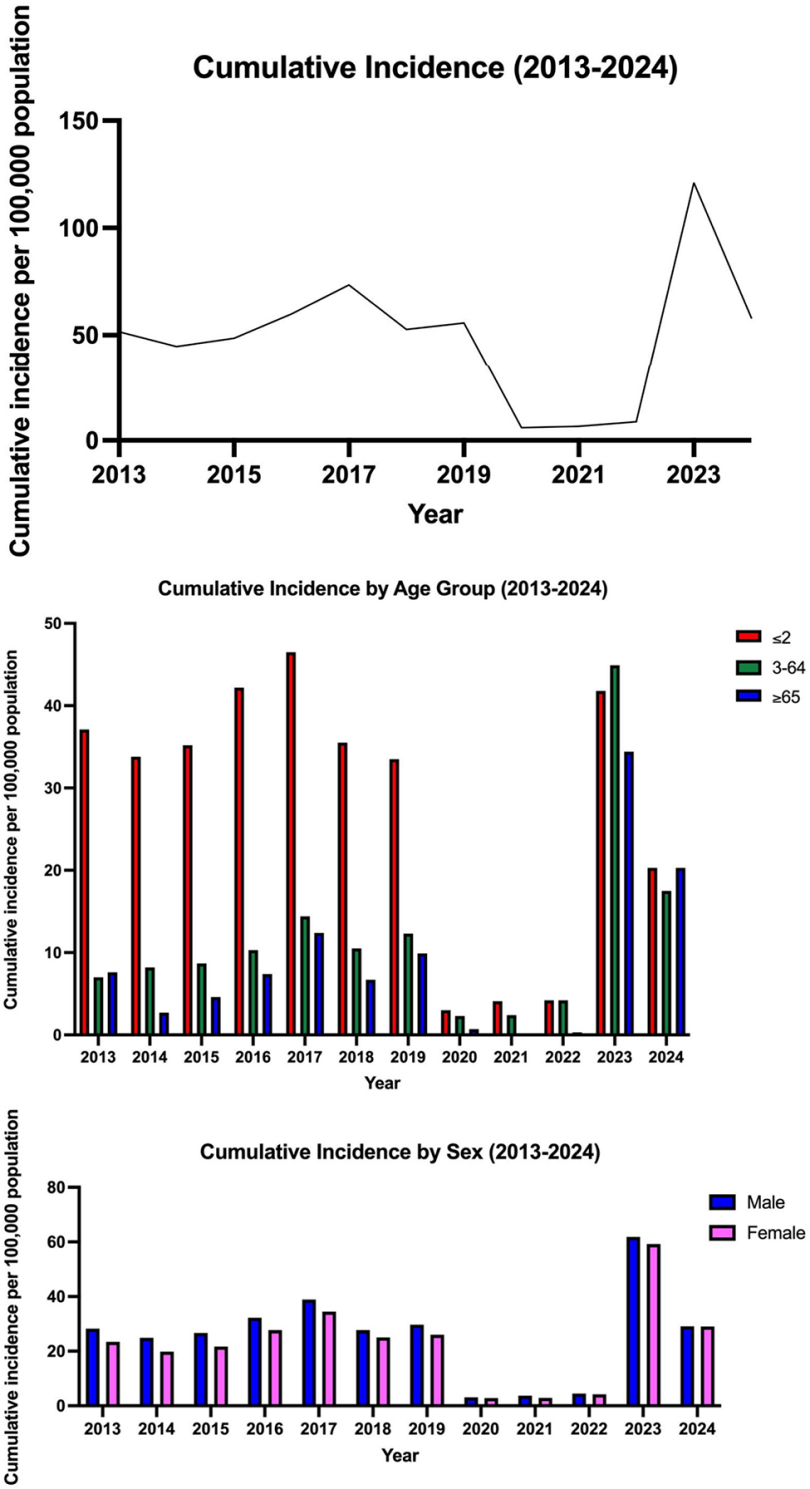
Cumulative Incidence (2013-2024)

**Methods:**

We conducted a retrospective population-based study using surveillance data from 2013 to 2024 in Hong Kong. RSV cases were extracted monthly, and the cumulative incidence (per 100,000 population) was calculated annually, stratified by age (≤2, 3–64, ≥65 years) and sex. Temporal trends, seasonal patterns, comorbidities, risk factors, and clinical outcomes (Intensive Care Unit (ICU) admissions, mortality, and ventilation usage) were examined. Seasonal-Trend decomposition using LOESS (STL) was applied under two scenarios:

1. Filtering out pandemic years (2020–2022) to create a discontinuous time series.
2. Including pandemic years (2020–2022) to analyze disruptions and recovery patterns.

**Figure:**
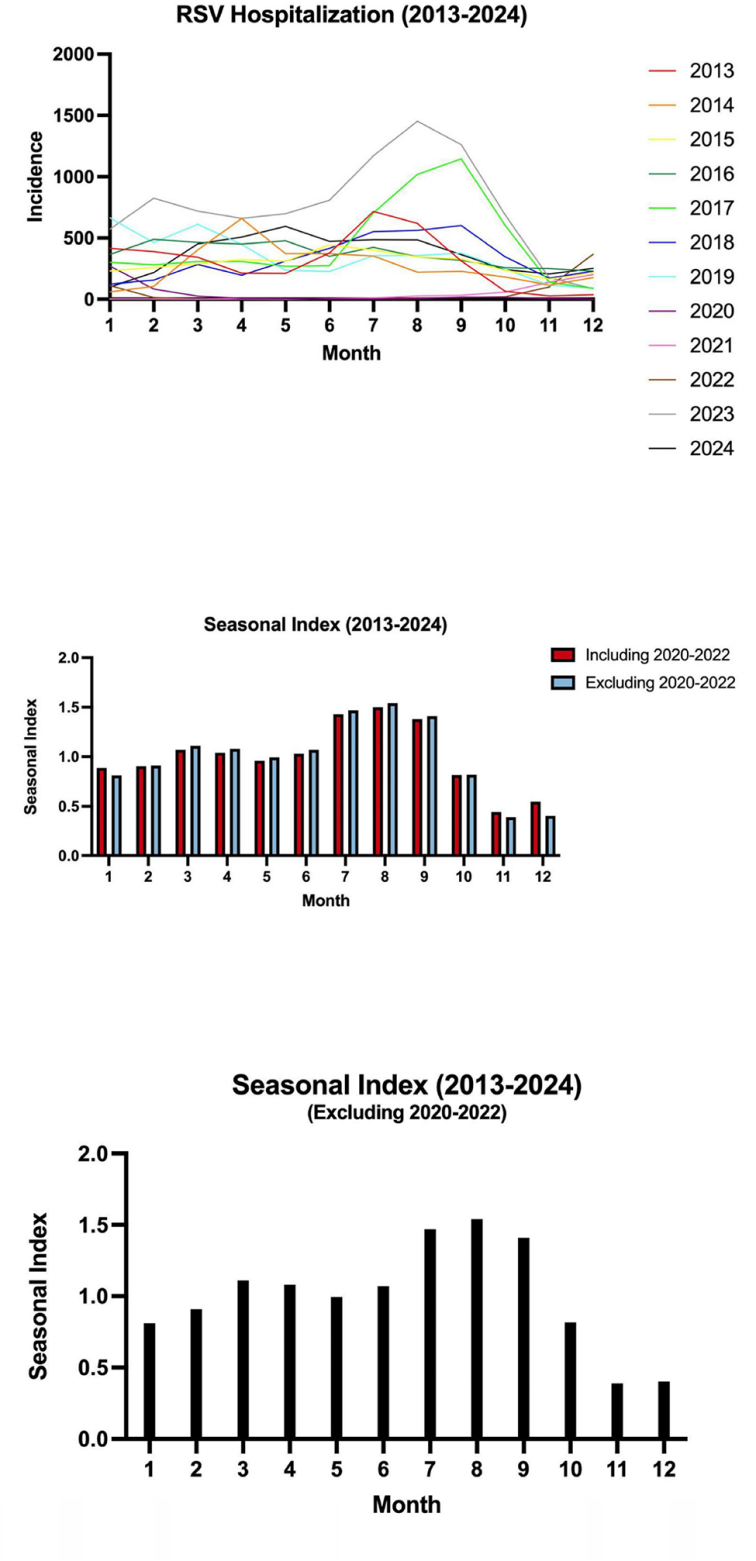
RSV Hospitalization and seasonal index (2013-2024)

**Figure:**
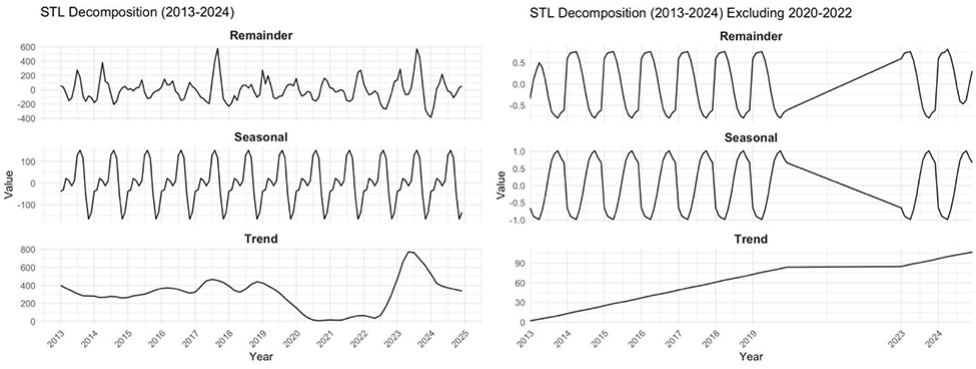
STL decomposition

**Results:**

Among 43,588 RSV cases, incidence peaked in 2017 (73.4/100,000) and 2023 (121.0/100,000). Children ≤2 years faced the highest burden (414.6/100,000 in 2023), while adults ≥65 years saw a 167% rise (7.6 to 20.3/100,000, 2013–2024). Seasonal peaks (July–September) were disrupted during COVID-19 but rebounded sharply in 2023, with severe outcomes: 253 ICU admissions and 186 deaths. STL decomposition showed pandemic-related suppression, followed by amplified seasonality post-COVID. Prematurity (10.3%) and chronic obstructive pulmonary disease (6.8%) were key risk factors.

**Conclusion:**

RSV remains a growing public health concern in Hong Kong, heavily impacting young children and older adults. The COVID-19 pandemic disrupted RSV transmission, but the post-pandemic resurgence was more intense. STL decomposition offered valuable insights into seasonal and temporal trends, highlighting the need for time-series modeling in predicting viral behavior. Enhanced surveillance, maternal vaccination, pediatric monoclonal antibodies, and targeted preparedness are critical to reducing RSV-related morbidity and mortality in high-risk groups.

**Disclosures:**

All Authors: No reported disclosures

